# Intuitionistic Fuzzy C-Means Algorithm Based on Membership Information Transfer-Ring and Similarity Measurement

**DOI:** 10.3390/s21030696

**Published:** 2021-01-20

**Authors:** Haipeng Chen, Zeyu Xie, Yongping Huang, Di Gai

**Affiliations:** 1 College of Computer Science and Technology, Jilin University, Changchun 130012, China; chenhp@jlu.edu.cn (H.C.); xiezy18@mails.jlu.edu.cn (Z.X.); gaidi18@mails.jlu.edu.cn (D.G.); 2 Key Laboratory of Symbolic Computation and Knowledge Engineering of Ministry of Education, Jilin University, Changchun 130012, China

**Keywords:** image segmentation, information transferring, fuzzy C-means algorithm, similarity

## Abstract

The fuzzy C-means clustering (FCM) algorithm is used widely in medical image segmentation and suitable for segmenting brain tumors. Therefore, an intuitionistic fuzzy C-means algorithm based on membership information transferring and similarity measurements (IFCM-MS) is proposed to segment brain tumor magnetic resonance images (MRI) in this paper. The original FCM lacks spatial information, which leads to a high noise sensitivity. To address this issue, the membership information transfer model is adopted to the IFCM-MS. Specifically, neighborhood information and the similarity of adjacent iterations are incorporated into the clustering process. Besides, FCM uses simple distance measurements to calculate the membership degree, which causes an unsatisfactory result. So, a similarity measurement method is designed in the IFCM-MS to improve the membership calculation, in which gray information and distance information are fused adaptively. In addition, the complex structure of the brain results in MRIs with uncertainty boundary tissues. To overcome this problem, an intuitive fuzzy attribute is embedded into the IFCM-MS. Experiments performed on real brain tumor images demonstrate that our IFCM-MS has low noise sensitivity and high segmentation accuracy.

## 1. Introduction

Recent years have witnessed the increasement of prevalence in glioma. The high incidence and mortality of glioma have threatened human health seriously. In clinical diagnosis, magnetic resonance technology provides an excellent assistance to medical treatment, which has the ability to detect brain tumors [1]. Therefore, segmenting tumor information in brain magnetic resonance images (MRI) by computer technology has become a current hot research field.

Nowadays, a variety of image segmentation techniques have been proposed, such as algorithms based on manual segmentation [2,3], boundary [4], atlas [5,6,7], kernel function [8,9,10,11], region growing technology [12,13,14,15,16] and clustering [17,18,19,20,21,22,23,24,25,26,27,28,29,30,31,32]. For the effectiveness and accuracy, the clustering-based segmentation has become the most popular method to classify different property elements. The algorithm based on clustering consists of two main types of architectures, including the soft and hard clustering-based segmentation methods. The information in an MRI has the characteristics of a complex structure and uneven gray distribution, which is difficult to segment. Conventional hard clustering-based segmentation often loses small-sized information, whereas the soft clustering-based algorithm achieves better properties in segmenting MRI information. Thus, in this paper, we will focus on MRI segmentation based on soft clustering. 

The fuzziness-based algorithms (FCM [23] and Intuitionistic Fuzzy C-means clustering (IFCM) [24]) assume that a pixel belongs to multiple clusters with different membership degrees [22]. All of them have the ability to achieve the soft segmentation of data and suit the complexity of medical images. For noise-free images, the FCM and IFCM can obtain well-pleasing segmentation results. Nonetheless, these two algorithms fail to combine neighborhood information, so that noise has a huge impact on their segmentation results. Consequently, how to ensure a segmenting brain tumor MRI accurately while enhancing the algorithm’s noise immunity has become a tough challenge. 

Recently, existing improved FCM can be roughly classified into three classes as follows: FCM improved by spatial constraint, kernel-based FCM and FCM based on neighborhood information.

In the spatial constraint-based FCM, the spatial constraint is merged into the objective function for robust clustering. For example, a conditional spatial fuzzy C-means clustering algorithm for the segmentation of an MRI (csFCM) [25] was presented, which took spatial constraint into the membership function. As a result, the robustness of the FCM to noise was enhanced. Based on the csFCM, a gamma correction conditional FCM algorithm with spatial information (GcsFCM) [26] was proposed, and the robustness was improved further. However, both the csFCM and GcsFCM are still sensitive to salt-and-pepper noise. Besides, an intuitionistic center-free FCM clustering for MR brain image segmentation (ICFFCM) [27] was applied for removing the noise. Although the robustness to noise is raised, the ICFFCM is more time-consuming because of its much-complicated calculation process of space constraints.

To achieve image denoising, the kernel-based FCM [8,9,10] mainly focuses on replacing the spatial distance information of the image by the kernel function. In the kernel-based FCM, a clustering of incomplete data using the kernel-based fuzzy C-means algorithm (KFCM) [8] was proposed to improve the robustness. Ulteriorly, the addition of the spatial penalty item makes the KFCM more suitable for the intensity unevenness of the MRI [9]. To reveal the non-Euclidean structure of the image, a new kernel-induced distance measure was combined with the FCM as the spatial constraints [10]. The authors considered a new kernel-based measurement method to obtain a new objective function to enhance the robustness further. However, the fuzzy clustering algorithm used for the brain MRI still lacks robustness to noise and outliers.

Many experiments [28,29,30,31,32] show that combining the neighborhood information is beneficial to improving the robustness. To obtain a robust clustering, Bai et al. merged the spatial information into the objective function [28], but it suffered defeat when classifying normal brain tissue. Using the neighborhood information, Lei et al. presented two FCM-based algorithms: one was a fast fuzzy C-means clustering algorithm based on superpixels [29], and the other was the improved FCM algorithm based on membership function filtering (FRFCM) [30]. In [29], the images were presegmented into several large patches, leading to a loss of the original details. Although the superpixel can adapt to the irregular image boundaries of a brain tumor MRI with effect [29], the images are pre-segmented in several large patches, leading to a loss of the original details. As a result, the superpixel-based FCM algorithm cannot segment brain tumor images as expected. Using the neighborhood information, the FRFCM enhanced the robustness to salt-and-pepper noise. However, it is less effective at detecting small-sized tumors. At the same time, an improved intuitionistic fuzzy C-means clustering algorithm that combines the local information for brain image segmentation (IIFCM) [31] was invented, but the segmentation accuracy was frustrating. Accordingly, how to improve the accuracy and robustness of the pixel-based FCM algorithm for a brain MRI has become a key issue for current research. 

In this paper, to overcome the defects of the algorithms as mentioned, we propose an intuitionistic fuzzy C-means algorithm based on membership filtering and similarity measurements, named the IFCM-MS. Without any preprocessing or postprocessing, the IFCM-MS achieves a low sensitivity to salt-and-pepper noise, as well as provides a high segmentation accuracy. Our main contributions can be summarized as follows:

- A membership information transfer model is proposed to fuse the information between the membership matrix generated by two adjacent iterations. In addition, the local neighborhood information is also considered during each iteration.
- We apply a similarity measurement method between pixels and cluster centers instead of simple distance measurements into the proposed IFCM-MS to modify the membership matrix. The local gray information and Euclidean distance information are both taken into consideration to integrate the location information and gray information. This property achieves a good enhancement of the segmentation ability.
- The intuitionistic fuzziness is embedded into the calculation process of similarity between the pixel and cluster centers, which achieves more accurate segmentation in the organization boundary.

The rest of this paper is organized as follows: In Section 2, we provide the materials for our algorithm. The method is described in Section 3. The experimental results are shown in Section 4, which also contains the discussion. Finally, we present our conclusion and future plans in Section 5.

## 2. Materials

### 2.1. Intuitionistic Fuzzy Set

In Zadeh’s fuzzy set theory [33], the value of membership is defined as the degree of elements belonging to each cluster center. Every element in the fuzzy set has a grade of membership in [0,1]. Mathematically, the fuzzy set *F* is expressed as

(1) $$F=\left\{\left(x,\mu_{F}(x)\right)|x\in E\right\}$$

where $\mu_{F}(x)$ represents the membership of each element *x* belonging to the full set *E*. For each *x*, the value of $\mu_{F}(x)$ is in [0,1].

Atanassov extended the fuzzy set and proposed the Intuitionistic Fuzzy Set (IFS) [34]. By adding uncertainty to the elements, IFS enhances the fuzziness of the description of objective things. The nonmembership of the original fuzzy set is a complement of the membership, but the nonmembership of the IFS is determined by the membership and hesitation of the element to the cluster. The mathematical expression of the IFS is

(2) $$IFS=\{(\mu_{IFS}(x),\nu_{IFS}(x),\pi_{IFS}(x))|x\in E\}$$

where $\mu_{IFS}(x)$ and $\nu_{IFS}(x)$ represent the fuzzy membership and nonmembership degree of the element belonging to the clusters, respectively. $\pi_{IFS}(x)$ denotes the uncertainty of the element to the clusters. To each x∈E, the range of $\mu_{IFS}(x)$, $\nu_{IFS}(x)$ and $\pi_{IFS}(x)$ is [0,1]; moreover, $\mu_{IFS}(x)+\nu_{IFS}+\pi_{IFS}(x)=1$.

The introduction of an intuitionistic fuzzy set adds an abundant uncertainty in the clustering process. Using this information, the algorithm will adequately accommodate the brain MRI segmentation.

### 2.2. Fuzzy C-Means Clustering Algorithm

The traditional FCM divides the pixels in the image into *c* numbers of clusters. Cluster centers and the membership of each element will be updated in each iteration, so as to minimize the objective function. By minimizing the objective function, the feature vectors are classified into different clusters. 

The objective function of the FCM is defined as

(3) $$J_{f}(U,V;X)=\sum_{i=1}^{a}\sum_{k=1}^{n}{\left(u_{ik}\right)}^{m}dis^{2}(x_{k},c_{i})$$

Equation (3) minimizes the objective function $J_{f}$ by updating the membership matrix *U* and the cluster center *V*, where $x_{k}$ is the *k*-th pixel in the image and $c_{i}$ is the *i*-th cluster center. $u_{ik}$ is the membership value, which can be calculated by evaluating the degree of membership between $x_{k}$ and $c_{i}$: $\sum_{i=1}^{a}u_{ik}=1$. The update method of $u_{ik}$ and $c_{i}$ is shown in Equations (4) and (5). $dis(x_{k},c_{i})$ is the Euclidean distance of $x_{k}$ with respect to the cluster center $c_{i}$.

(4) $$c_{i}=\frac{\sum_{k=1}^{n}\left(x_{k}u_{ik}^{m}\right)}{\sum_{k=1}^{n}u_{ik}^{m}}$$

(5) $$u_{ik}=\frac{{\left\|x_{k}-c_{i}\right\|}^{-2/(m-1)}}{\sum_{j=1}^{a}{\left(\left\|x_{k}-c_{j}\right\|\right)}^{-2/(m-1)}}$$

Although the FCM is widely used in medical image segmentation, Equation (3) shows that there is no neighborhood information in clustering processing. Besides, the lack of local spatial information results in an unsatisfactory robustness to noise, which leads to a poor outcome. 

### 2.3. Cluster–Center-Free Reformulation of the FCM

To reduce the noise sensitivity of the FCM, the cluster–center-free reformulation of the FCM takes advantage of hierarchical clustering [35] and aims to measure the location similarity between the pixels and clusters. The objective function of the reformulated FCM is given in Equation (6):

(6) $$J_{FCM\_CenterFree}(U,V;X)=\sum_{i=1}^{c}\sum_{k=1}^{n}{\left(u_{ik}\right)}^{m}\left(\frac{1}{{\left(\rho_{ik}\right)}^{2}}\right)$$

(7) $$\rho_{ik}=\frac{\sum_{j=1}^{n}{\left(u_{ij}\right)}^{m}w_{kj}}{\sum_{j=1}^{n}{\left(u_{ij}\right)}^{m}}$$

(8) $$w_{kj}=\exp\left(-\frac{{\left(dis(x_{k},x_{j})\right)}^{2}}{\alpha^{2}*\underset{\forall k,\forall j}{\max^{2}}\left(dis(x_{k},x_{j})\right)}\right)$$

where *c* is the number of clusters, *n* is the number of pixels in the image and $dis(x_{k},x_{j})$ represents the space distance between two pixels. In particular, $\rho_{ik}$ is the similarity between $x_{k}$ and the *i*-th cluster. $w_{kj}$ is the comparability between every two pixels, and *α* is the parameter, *α* > 0. 

Usually, the Lagrange method is adopted to minimize the objective function in the FCM system. So, Equation (6) can be minimized under the conditions of $\sum_{i=1}^{c}u_{ik}=1$, and the value in Equation (6) can be obtained as:

(9) $$u_{ik}=\frac{{\left(\rho_{ik}\right)}^{2/(m-1)}}{\sum_{i=1}^{c}{\left(\rho_{ik}\right)}^{2/(m-1)}}$$

According to Equation (9), the cluster centers are not updated in the cluster–center-free FCM, but the positional similarity between the pixel and clusters is adopted instead of the Euclidean distance, which improves the robustness of the algorithm. However, the cluster–center-free FCM only focuses on the spatial location information of the pixels; the gray information between the pixels and clusters is ignored, leading to the failure to use the local spatial information. As a consequence, cluster–center-free FCM cannot obtain excellent results when the medical images are segmented with serious noise pollution.

### 2.4. Fast-Guided Filter

The fast-guided filter is an upgrade of the guided filter, which was wildly applied in real products long ago. The guided filter can be driven as a linear model as follows:

(10) $$q_{i}=a_{k}I_{i}+b_{k},\;\forall i\in windows_{k}$$

(11) $$a_{k}=\frac{\frac{1}{\left|win\right|}\sum_{i\in win_{k}}I_{i}p_{i}-\mu_{k}{\bar{p}}_{k}}{\sigma_{k}^{2}+\varepsilon }$$

(12) $$b_{k}={\bar{p}}_{k}-a_{k}\mu_{k}$$

where the guidance image, filtering input image and filtering output image are expressed as *I*, *p* and *q*, respectively; *i* is the current pixel and *k* is the index of a local window *win* with a radius *r*. For suppressing the error between *p* and *q*, $a_{k}$ and $b_{k}$ are two parameters that can be calculated as Equations (11) and (12). $\mu_{k}$ and $\sigma_{k}$ are the mean and variance of image *I* in window *k*; additionally, ε is a parameter controlling the smoothing degree. The output image is computed by Equation (13):

(13) $$q_{i}={\bar{a}}_{i}I_{i}+{\bar{b}}_{i}$$

where ${\bar{a}}_{i}$ and ${\bar{b}}_{i}$ are the average of *a* and *b*, respectively, on the $win_{i}$ centered at *i*. The major computation is for ${\bar{a}}_{i}$ and ${\bar{b}}_{i}$. The fast-guided filter enhances the guided filter; firstly, the input *p* and guidance *I* are subsampled (nearest-neighbor or bilinear) by the ratio *s*. Then, ${\bar{a}}_{i}$ and ${\bar{b}}_{i}$ can be calculated, respectively, by Equations (11) and (12). Finally, the output image $q_{i}$ will be produced in accordance with Equation (13), which is up-sampled for *s* times to contain the same size as the original image.

## 3. Method

### 3.1. Overview

**Figure 1:**
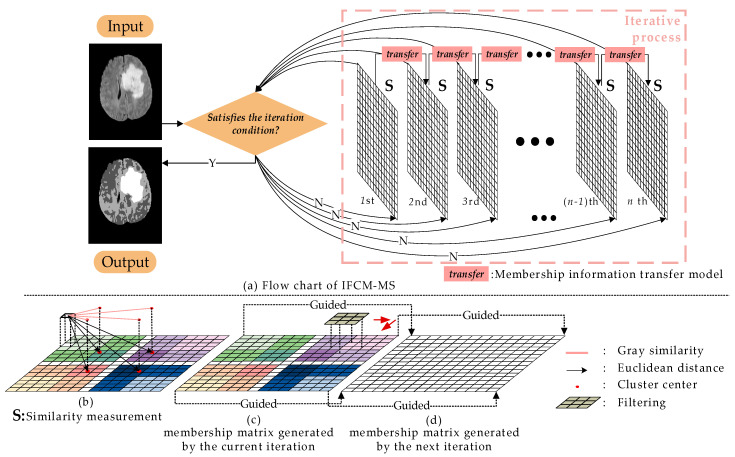
Overall flow chart. (**a**) Flow chart of IFCM-MS. (**b**) Similarity measurement. (**c**) Membership matrix generated by the current iteration. (**d**) Membership matrix generated by the next iteration. (**b**,**c**) Membership information transfer model.

In order to overcome the shortcoming that conventional FCM is sensitive to noise, we proposed the IFCM-MS. The specific process of IFCM-MS is shown in Figure 1.

In Figure 1, the membership matrixes generated by each iteration are represented as the grids, and the number of iterations are marked below the grids. Figure 1a stands for the similarity measurement method, while Figure 1b,c are the membership information transfer model. In Figure 1a,b, different clusters are expressed as different color systems, and the intensity of the color represents the degree of membership to the clustering center. Based on Figure 1, the overall process of the IFCM-MS can be generalized as follows:

- Membership information transfer model

There is no correlation between two iterations in the original FCM. As a result, the FCM is sensitive to noise. To address this problem, we introduce the membership information transfer model into the FCM. In the clustering process of the IFCM-MS, the membership information transfer model is inserted into the neighboring membership matrixes for transmitting information after each iteration, eliminating the influence of noise and protecting the detailed features of medical images.

- Similarity measurement method

We apply the similarity measurement method to calculate the membership matrix, so that the segmentation ability and robustness of the IFCM-MS are both improved. Specifically, the simple Euclidean distance between the pixel and cluster centers is replaced by the grayscale similarity and the degree of distance proximity.

- Clustering

To obtain a more accurate segmentation result, the IFCM-MS embeds the intuitive fuzzy attribute into the clustering process. Moreover, the IFCM-MS achieves a robust segmentation of noisy images without any preprocessing or postprocessing.

### 3.2. Membership Information Transfer Model

Generally, the addition of local information is effective in eliminating the influence of noise. However, the underutilization of the neighborhood information does not obtain satisfactory results. Therefore, in this paper, the membership information transfer model is inserted between the next two iterations, which reduces the impact of noise, as well as exploits the local spatial information.

Since the fast guided filter has a satisfactory effect on restoring the image edge and detail information, the median filter is also adept at smoothing noise; hence, the advantages of these two filters are fully integrated into the IFCM-MS. Different from directly filtering the image, the presented membership information transfer model will be adapted into the iterative process for restoring the membership matrix. According to Equations (11)–(13), the membership information transfer is considered as

(14) $$U_{q}={\bar{a}}_{i}U_{i}+{\bar{b}}_{i}$$

(15) $$a_{i}=\frac{\frac{1}{\left|win\right|}\sum_{k\in win_{i}}U_{pre}U_{k}-\mu_{i}{\bar{U}}_{i}}{\sigma_{i}^{2}+\varepsilon },\;U_{i}=med\{U_{pre}\}$$

(16) $$b_{i}={\bar{U}}_{i}-a_{i}\mu_{i}$$

where $U_{i}$, $U_{k}$ and $U_{q}$ denote the guidance matrix, filtering input matrix and filtering output matrix, respectively, and $U_{pre}$ is the membership matrix produced by the last iteration. Operation *med*{} represents the median filtering. Other variables have the same meaning as the fast-guided filter.

The traditional fast-guided filter requires a guidance image to repair the image. Usually, the guidance image is the same as the input, which results in the residue of noise. To address this problem, we took advantage of the present membership matrix as the input matrix. After median filtering, the matrix generated in the last iteration is embedded into the current processing as the guiding matrix. The successive transfer of information between two adjacent membership matrixes repairs the clustering results layer-by-layer.

### 3.3. Similarity Measurements

Based on the Euclidean distance, the traditional FCM integrates the distance information of a pixel with respect to the cluster centers into the clustering process, thus realizing the soft segmentation. However, the lack of gray information makes the FCM poor for noise-contaminated image segmentation. In Section 3.2, we combine the neighborhood information of each pixel while contacting the adjacent iteration information, but the information of the single pixels is not fully utilized. Therefore, to improve the segmentation accuracy and reduce the noise sensitivity of the IFCM-MS, we further combine the gray information of the pixel, thereby proposing a similarity measurement method.

In the IFCM-MS, the similarity of the gray and distance between the pixel and cluster center are both considered in our method. In the calculation of the membership matrix for each pixel, we design two adaptive parameters so that the weight of the distance information and gray information can be adjusted automatically. As the noise is expressed as regional extreme points, when updating the membership matrix, the noise points with high-grayscale similarity but low-distance similarity from the cluster center will not be classified into the same category. The similarity measurement method is defined as follows:

(17) $$Sim\left\|x_{k},c_{i}\right\|=w_{d}(x_{k},c_{i})*w_{g}(x_{k},c_{i})$$

where $Sim\left\|x_{k},c_{i}\right\|$ is the similarity between pixel $x_{k}$ and cluster center $c_{i}$, and $w_{d}$ and $w_{g}$ denote the distance similarity and gray similarity, respectively. By introducing two adaptive parameters, *D* and *G*, we obtain $w_{d}$ and $w_{g}$ as follows:

(18) $$w_{d}=\exp\left(-\frac{dis^{2}(x_{k},c_{i})}{D}\right)$$

(19) $$w_{g}=\exp\left(-{\left(\frac{g\left(x_{k}\right)-g\left(c_{i}\right)}{G}\right)}^{2}\right)$$

where $dis^{2}(x_{k},c_{i})$ is the Euclidean distance between $x_{k}$ and $c_{i}$, and *g* represents the gray of pixel. 

Since the mean deviation could reflect the distribution of the data, in Equations (18) and (19), *D* and *G* are designed to be:

(20) $$D=\frac{1}{n}\sum_{i=1}^{n}\left(dis(x_{k},c_{i})-\frac{1}{n}\sum_{i=1}^{n}dis(x_{k},c_{i})\right)$$

(21) $$G=\frac{1}{n}\sum_{i=1}^{n}\left({\left[g\left(x_{k}\right)-g\left(c_{i}\right)\right]}^{2}-\frac{1}{n}\sum_{i=1}^{n}{\left[g\left(x_{k}\right)-g\left(c_{i}\right)\right]}^{2}\right)$$

With the decrease of the spatial distance between the pixel and cluster center, the contribution of $w_{d}$ would increase, which means the pixel adjacent to the cluster center has a larger weight. Meanwhile, if a pixel is not only near to a cluster center but also owns a smaller gray variance (a larger $w_{g}$), the value of $Sim\left\|x_{k},c_{i}\right\|$ would be larger as well. As a result, the pixel would be considered as a member of the cluster.

By the similarity measurement method, the gray information and spatial relation are both adopted into the membership matrix calculation. Ulteriorly, the segmentation ability and robustness of the IFCM-MS is upgraded. Additionally, the overall calculation of the IFCM-MS will be discussed in Section 3.4.

### 3.4. Clustering

The intuitionistic fuzzy set is proven suitable for medical image segmentation. Therefore, aiming to deal with the complex structure of brain medical images, the intuitionistic fuzzy attribute is adopted into the clustering process of the IFCM-MS.

From the perspective of the objective function, the embedding of the IFS is manifested as the addition of intuitionistic fuzzy entropy ($\sum_{i=1}^{a}{\pi^{\prime }}_{i}e^{1-{\pi^{\prime }}_{i}}$, ${\pi^{\prime }}_{i}=\left(1/n\right)\sum_{k=1}^{n}\pi_{ik}$, *k* ∈ [1,*n*]). The intuitionistic fuzzy entropy will calculate the hesitation, which exploits more information. It is clear that the IFS is more appropriate for segmenting the medical images with complex distributions and fuzzy edges.

According to Equations (3) and (6), the objective function of the IFCM-MS is:

(22) $$J(U,V;\xi )=\sum_{i=1}^{a}\sum_{k=1}^{n}{\left({u^{\prime }}_{ik}\right)}^{m}\left(\frac{1}{S_{ik}^{2}}\right)+\sum_{i=1}^{a}{\pi^{\prime }}_{i}e^{1-{\pi^{\prime }}_{i}},m=2$$

(23) $$S_{ik}=\frac{\sum_{k=1}^{n}{\left({u^{\prime }}_{ik}^{}\right)}^{m}Sim\left\|x_{k},c_{i}\right\|}{\sum_{k=1}^{n}{\left({u^{\prime }}_{ik}^{}\right)}^{m}},m=2$$

where ${u^{\prime }}_{ik}$ is the intuitionistic fuzzy membership value of pixel $x_{k}$ to cluster center $c_{i}$: ${u^{\prime }}_{ik}=u_{ik}+\pi_{ik}$. Moreover, when the membership degree generated is calculated again in the next iteration for transferring the information, the current ${u^{\prime }}_{ik}$ will be utilized as a member of the guided matrix to the next iteration.

The LaGrange multiplier method can be applied to minimize the objective function under constraint condition $\sum_{i=1}^{a}{u^{\prime }}_{ik}=1$:

(24) $$\tilde{J}=\sum_{i=1}^{a}\sum_{k=1}^{n}{\left({u^{\prime }}_{ik}\right)}^{m}\left(\frac{1}{S_{ik}^{2}}\right)+\sum_{i=1}^{a}{\pi^{\prime }}_{i}e^{1-{\pi^{\prime }}_{i}}+\lambda \left(\sum_{i=1}^{a}{u^{\prime }}_{ik}-1\right)$$

We calculate the minimum value of the partial differential equation by using the partial derivative of ${u^{\prime }}_{ik}$ and $v_{i}$:

(25) $$\frac{\partial \tilde{J}}{\partial {u^{\prime }}_{ik}}=\frac{\partial \left[\sum_{i=1}^{a}\sum_{k=1}^{n}{\left({u^{\prime }}_{ik}\right)}^{m}\left(1/S_{ik}^{2}\right)+\sum_{i=1}^{a}{\pi^{\prime }}_{i}e^{1-{\pi^{\prime }}_{i}}\right]}{\partial {u^{\prime }}_{ik}}+\lambda$$

Then, let

(26) $$\frac{\partial \tilde{J}}{\partial {u^{\prime }}_{ik}}=\sum_{i=1}^{a}\sum_{k=1}^{n}m{\left({u^{\prime }}_{ik}\right)}^{m-1}\left(1/S_{ik}^{2}\right)+\lambda =0$$

(27) $$\frac{\partial \tilde{J}}{\partial \lambda }=\sum_{i=1}^{a}{u^{\prime }}_{ik}-1=0$$

Solving Equation (26), we get ${u^{\prime }}_{ik}$:

(28) $${u^{\prime }}_{ik}=-\frac{\lambda^{1/(m-1)}}{m\sum_{i=1}^{a}{\left(1/S_{ik}^{2}\right)}^{1/(m-1)}}$$

Substituting Equation (28) into Equation (26), we get

(29) $$\lambda =-2m(1/S_{ik}^{2})$$

So, the membership update formula is:

(30) $${u^{\prime }}_{ik}=\frac{{\left(1/S_{ik}^{2}\right)}^{-1/(m-1)}}{\sum_{i=1}^{a}{\left(1/S_{ik}^{2}\right)}^{-1/(m-1)}}$$

According to Equation (4), the cluster center update formula is obtained as

(31) $$v_{i}=\frac{\sum_{k=1}^{n}{u^{\prime }}_{ik}x_{k}}{\sum_{k=1}^{n}{u^{\prime }}_{ik}}$$

With the ${u^{\prime }}_{ik}$ in Equation (31), the objective function *J* could be minimized. According to Equations (14)–(31), the steps of the IFCM-MS can be summarized in Algorithm 1.
**Algorithm 1:** Flow of the proposed algorithm. IFCM-MS: intuitionistic fuzzy C-means algorithm based on membership information transferring and similarity measurements**Input:** Noise image**Output:** Segmented tumor image**Set parameters:** the number of clustering center *n*, the number of pixels *N*, iteration parameter *m*, the maximum number of iterations *a* and the minimums error value of the objective function *η*.1:**Initialize:** generate the initial membership matrix U randomly, $x_{k}$ represents pixel, *φ* is clustering center calculated currently, $w_{d}$ and $w_{g}$ are the distance similarity and gray similarity respectively, *D* and *G* are two adaptive parameters based on location and grat difference, variable $Sim\left\|x_{k},c_{i}\right\|$ is the similarity, ${\bar{a}}_{i}$ and ${\bar{b}}_{i}$ are two parameters that guide the membership matrix $U_{q}$.2:**for***j* = 1 to *a*
**do**3: **for**
*k* = 1 to *N*
**do**4:  **for**
*i* = 1 to *n*
**do**5:   Calculate D and G by Equations (20) and (21)6:   Calculate $w_{d}$ and $w_{g}$ by Equations (18) and (19)7:   
$Sim\left\|x_{k},c_{i}\right\|=w_{d}(x_{k},c_{i})*w_{g}(x_{k},c_{i})$
8:   
$S_{ik}=\frac{\sum_{k=1}^{n}{\left({u^{\prime }}_{ik}^{}\right)}^{m}Sim\left\|x_{k},c_{i}\right\|}{\sum_{k=1}^{n}{\left({u^{\prime }}_{ik}^{}\right)}^{m}}$
9:   
${u^{\prime }}_{ik}=\frac{{\left(1/S_{ik}^{2}\right)}^{-1/(m-1)}}{\sum_{i=1}^{a}{\left(1/S_{ik}^{2}\right)}^{-1/(m-1)}}$
10:   
$v_{i}=\left(\sum_{k=1}^{n}{u^{\prime }}_{ik}x_{k}\right)/\left(\sum_{k=1}^{n}{u^{\prime }}_{ik}\right)$
11:  **end for**12: **end for**13: 
$U_{q}={\bar{a}}_{i}U_{i}+{\bar{b}}_{i}$
14: **if** {$U_{q}-U_{i}\leq \eta$} **then**
*break*15: **end if**16:**end for**

## 4. Results and Discussion

### 4.1. Environment Settings and Evaluation Index 

To estimate the effectiveness and robustness of the IFCM-MS, the real brain tumor MRI of 25 patients in the BRATS 2012 dataset were tested in our experiments. All the images were detected through the FLAIR modality. In order to prove the nonspeculative nature of the algorithm for each individual, one to two images adjacent or similar to each patient were selected. The weighting exponent was the same as the IFCM: *m* = 2, *η* = 10^−5^. Five state-of-the-art clustering algorithms: FCM, IFCM, sFCM, csFCM and FRFCM were employed in these experiments to compare with the IFCM-MS. These algorithms have different advantages: FCM, IFCM and FRFCM have high accuracy, while sFCM, csFCM and FRFCM have a strong capability of noise removal.

All the experimental methods in this paper were performed using MATLAB R2018a installed on the 64-bit Windows 10 operating system (Intel^®^Core i5-8300H), and the CPU was equipped with a 2.30 GHz processor and 8 GB RAM.

In this paper, four performance measures: accuracy, precision, specificity and recall were used to evaluate the segmentation quantitatively. Accuracy is one of the most commonly used evaluation indicators in the field of segmentation and detection, which indicates the degree of overlap between the segmentation result and ground truth. A larger accuracy value expresses a more accurate segmentation result. Precision assesses the proportion of pixels in the results classified correctly, which means the number of true positives (*TP*). The greater the number of *TP*, the higher the value of precision. Specificity evaluates the proportion of pixels in the results classified falsely, which means the number of false positives (*FP*). A higher *FP* number will result in a low specificity value. Recall is the ratio of *TP* to all positive values (the sum of the *TP* and false negatives (*FN*) [36,37]). The specific calculation methods of the four indicators are as follows:

(32) $$accuracy=\frac{TP+TN}{TP+TN+FP+FN}$$

(33) $$precision=\frac{TP}{TP+FP}$$

(34) $$recall=\frac{TP}{TP+FN}$$

(35) $$specificity=\frac{TN}{TN+FP}$$

where *TN* represents the quantity of pixels belonging to the background area in the segmentation results and ground truth. *FN* represents the number of pixels in the tumor area of the segmentation results that do not belong to the region of interest in the ground truth.

### 4.2. Ablation Study

Image segmentation plays a crucial role in medical diagnosis. It is always challenging to segment a medical image because of its complexities, such as noise, blur and intensity nonuniformity. To demonstrate the superiority of the IFCM-MS, the brain tumor medical images are utilized as test images in this section.

To exhibit the gradual improvement process of the algorithm, the ablation experiment is applied into our assignment to verify the effectiveness of the algorithm.

The IFCM-MS mainly enhances the FCM through three levels:

- Neighborhood Information:

The membership information transfer model not only combines the neighborhood information of pixels into the clustering process but also completes the information transfer between two adjacent iterations.

- Spatial Information:

The original FCM only combines the Euclidean distance information, which leads to poor segmentation results in noisy image segmentation. Based on the Euclidean distance, the similarity measurements take the gray difference between the pixel and cluster center into account, which further upgrades the robustness of the IFCM-MS.

- Adaptive Evolution:

The embedding of the intuitionistic fuzzy attribute improves the adaptability of the IFCM-MS to the brain medical images.

This section will add the above three levels sequentially on the basis of the original FCM, and experimental data is provided, respectively, to reveal the improvement process of the IFCM-MS.

**Figure 2:**
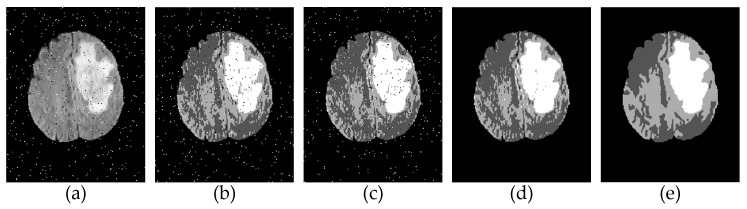
Step-by-step results of the intuitionistic fuzzy C-means algorithm based on membership filtering and similarity measurements (IFCM-MS). (**a**) Original image. (**b**) Fuzzy C-means algorithm (FCM). (**c**) FCM + intuitionistic fuzzy set (IFS). (**d**) FCM + IFS + membership information transfer model. (**e**) FCM + IFS + membership information transfer model + similarity measurements.

Figure 2 provides the results generated by each step of the evolution from the conventional FCM to the IFCM-MS. In Figure 2b, the tumor is classified as normal brain tissue, and the result is heavily affected by noise. After the addition of the IFS, as Figure 2c shows, the misclassified area is adjusted, but the influence of noise is not eliminated. On the basis of Figure 2c, Figure 2d is embedded with the membership information transfer model, which ensures the correct classification of pixels while overcoming the noise. However, the scattered points due to misclassification still exist in Figure 2d. Figure 2e not only reduces the noise sensitivity but also eliminates the scattered points and improves the accuracy of the segmentation. According to the above analysis, it can be seen that our methods are valid in the process of improving the original FCM.

In order to quantifiably discuss the improvement process of the FCM, the objective data analysis of each improvement result is listed in Table 1 and Table 2. What’s more, the best score has been marked.

Table 1 exhibits the ablation experiment results of Figure 2 under the salt-and-pepper noise with 0.02 intensity. To eliminate the speculative nature of the ablation experiments, under the same conditions, the average values of all the experimental results are listed in Table 2.

Except for showing the evaluation index scores of the results in each step, the best scores were marked in Table 1 and Table 2. It can be seen from Table 1 and Table 2 that, in any evaluation index, our method achieves the upgrade of segmentation accuracy and robustness in every step of the evolution. 

### 4.3. Subjective Evaluation of Experimental Results

The FCM and IFCM are able to segment a brain tumor MRI with a good result, but the local spatial information is neglected. Therefore, the FCM and IFCM are powerless to resist noise-polluted images. In our work, a membership information transfer model is proposed in the IFCM-MS to fuse adjacent iteration information and matrix neighborhood information; gray difference and distance information are both used in the similarity measurements to introduce the local spatial information; moreover, the design of two adaptive parameters makes the measurement method more flexible, and the intuitive fuzzy attribute is employed for enhancing the algorithm’s ability to segment medical images.

**Figure 3:**
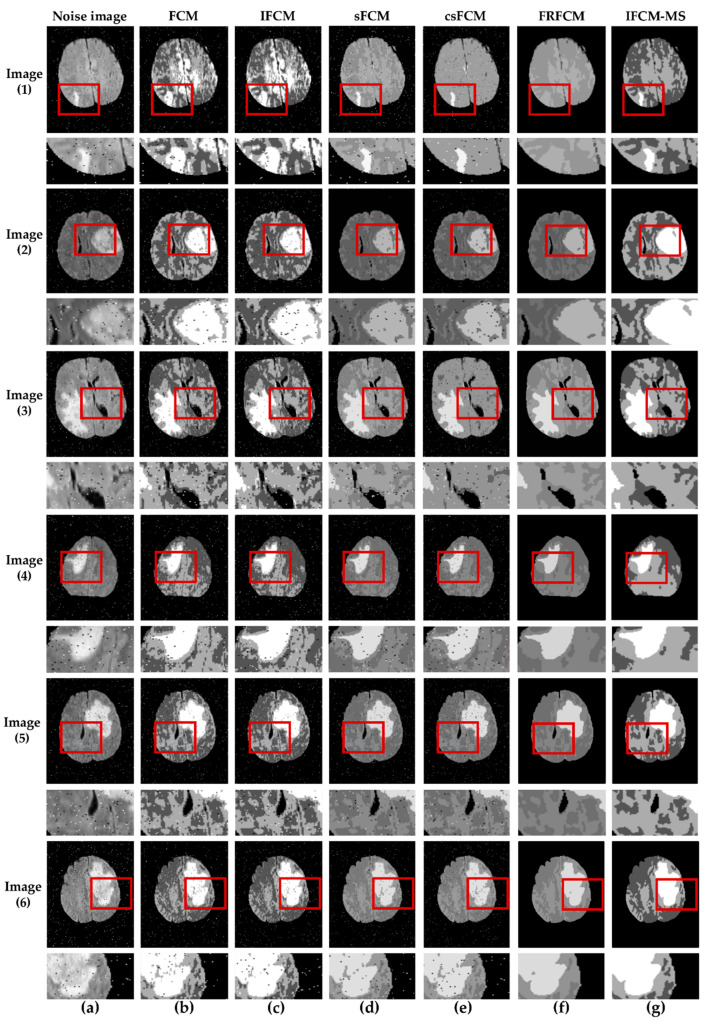
Segmentation results of noisy images (all the areas marked by red squares were enlarged and placed below the corresponding image). (**a**) Noise image. (**b**) FCM. (**c**) IFCM. (**d**) sFCM: spatial fuzzy C-means clustering algorithm. (**e**) csFCM: conditional spatial fuzzy C-means clustering algorithm. (**f**) FRFCM: FCM algorithm based on membership function filtering. (**g**) IFCM-MS.

To verify the segmentation performance of the IFCM-MS further, we added 2% of salt-and-pepper to the images. Moreover, the proposed algorithm is compared with the other algorithms described above. Figure 3 shows the segmentation results of the tumor produced by the different algorithms mentioned above. In Figure 3, the results are marked by red squares; for facilitating observations, the marked area was enlarged and placed below the original image. It is clear that our algorithm shows excellent performance in tumor detection. As the images of Figure 3b,c indicate, the FCM and IFCM do not overcome its sensitivity to noise, which leads to poor segmentation results. More than that, the tumor is clustered into normal brain tissue. Though the csFCM combines the spatial neighborhood information, it is unable to segment the tumor efficiently. In addition, the results from the csFCM segment the normal brain tissue inaccurately (red squares of column (e)). Just like the red squares show, for Image (2), there are many misclassifications of the tumor area in Image (2b–f), whereas the IFCM-MS provides good segmentation results (Image (2g)). Compared with the FCM and IFCM, the results from the IFCM-MS and FRFCM eliminated the noise. However, it is obvious that the results from the IFCM-MS provide unambiguous internal organization and maximum robustness, which signifies the best effect for tumor segmentation. 

To test the ability of the IFCM-MS in small-sized tumor detection, Figure 3 lists the segmentation results of Image (1). From a subjective judgment, we can see that many misclassifications exist in Image (1b,c); although the tumor is located in Image (1d,e), it is greatly affected by noise and suffers a defeat in classifying the normal brain tissue. Image (1f) shows that the FRFCM fails to detect a small-sized tumor. In contrast, the IFCM-MS still provides a better effect in the detection of the small-sized tumor.

The IFCM-MS provides better segmentation results than other algorithms due to the introduction of the membership information transfer model and similarity measurement method. After three-dimensional (3D) reconstruction technology, the segmentation results can estimate the shape and volume of the tumor, by which doctors can make an appropriate medical plan.

**Figure 4:**
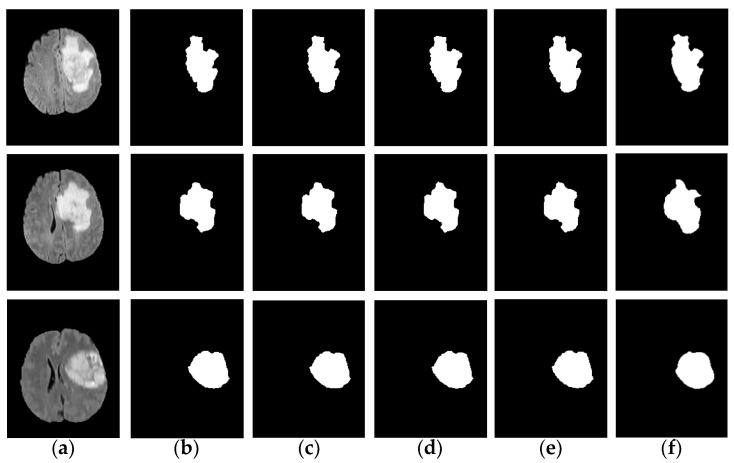
Tumors extracted under different noise intensities. (**a**) Original image. (**b**–**e**) Results (the noise intensities are 0, 0.005, 0.01 and 0.02, respectively). (**f**) Ground truth.

In order to verify the excellent performance of the IFCM-MS from all aspects, experiments on robustness were also added to our assignment: we used the IFCM-MS to segment the tumor area in the noisy images with different intensities, as shown in Figure 4. Figure 4b–e is the tumor information extracted when the noise intensity is 0, 0.005, 0.01 and 0.02. Figure 4f is the ground truth. Compared with ground truth, the IFCM-MS is equipped to obtain good segmentation results under different noise intensities and shows a strong robustness to salt-and-pepper noise. Moreover, to verify the effective performance of the IFCM-MS, the objective discussions of the segmentation results corresponding to each method on the BRATS 2012 dataset will be reported in Section 4.4.

### 4.4. Objective Discussion on Robustness and Accuracy

In the cases of 0% and 2% noise intensities, the average and STD values of accuracy, precision, specificity and recall on BRATS 2012 are given in Table 3, and the optimal values for each noise intensity are marked respectively (0: bold and italics, 0.02: bold). After comparing the results of each algorithm with the ground truth, it can be found that the IFCM-MS consistently performed well in accuracy, specificity and recall. However, there are morphological operations in FRFCM such as preprocessing; moreover, postprocessing is consistent in sFCM and csFCM. As a result, the precision of the IFCM-MS is slightly lower than the other comparison algorithms. 

**Figure 5:**
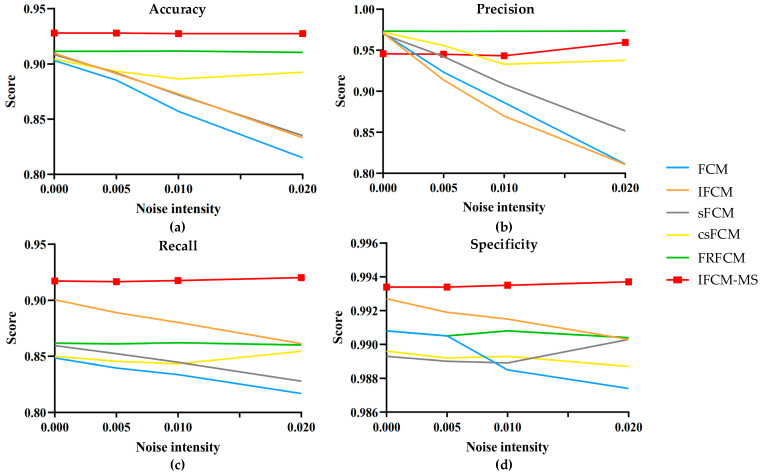
The change of each index value under different noise intensities. (**a**) Accuracy. (**b**) Precision. (**c**) Recall. (**d**) Specificity.

In addition, salt-and-pepper noise was added with intensities of 0, 0.005, 0.01 and 0.02 to the test images. The evaluation indicators of the IFCM-MS and comparison algorithms were calculated. The statistical results are shown in Figure 5.

Figure 5a–d represents the fluctuation range of the evaluation indicators accuracy, precision, recall and specificity, respectively. The vertical axis represents the average evaluation value of all the experimental results, and the horizontal axis expresses the different noise intensities. As shown in Figure 5, compared with the FCM, IFCM, sFCM, csFCM and FRFCM, not only the accuracy, recall and specificity of IFCM-MS are closer to 1 but, also, the fold line fluctuation range is the lowest. Therefore, it is obvious that the IFCM-MS is much more robust and competent than the other algorithms. 

There is no preprocessing (e.g., direct filtering and morphological operation) and postprocessing in the IFCM-MS; as a result, the precision index of the IFCM-MS is slightly lower than the csFCM and FRFCM. However, the csFCM does not overcome its sensitivity to noise, and the FRFCM obtains a poor segmentation result with small-sized tumors. The variation range of the IFCM-MS is still more stable than the other algorithms. Moreover, the most critical accuracy is much higher than the other algorithms. 

From the experiments and discussion above, the IFCM-MS has the capacity to provide good results for brain tumor medical images. Simultaneously, it has a better performance than the other algorithms, especially for accuracy, recall and specificity. The main reason is that the proposed IFCM-MS takes full advantage of local spatial information and adopts the intuitive fuzzy attribute to enhance the segmentation ability. 

Consequently, the IFCM-MS is superior in both noise-immunity and segmentation-accuracy while ensuring the segmentation performance of the brain MRI.

## 5. Conclusions

Aiming at the brain tumor MRI segmentation, an intuitionistic fuzzy C-means algorithm based on membership information transferring and similarity measurements was proposed, namely the IFCM-MS. By introducing the membership information transfer model, the neighborhood information and adjacent membership matrixes information were both exploited in clustering. Since the robustness of the IFCM-MS can be enhanced by combining these two types of information, it is easy to achieve detail preservation and noise elimination simultaneously. Moreover, the IFCM-MS employed a similarity measurement method to improve the segmentation accuracy. Except for considering the Euclidean distance, this new mixed measurement fused the gray information adaptively. Furthermore, we embedded an intuitive fuzzy attribute into the clustering process, which can adapt to the fuzziness in medical images perfectly. In addition, by the ablation experiment, we demonstrated that our method enhanced the FCM both the robustness and accuracy aspects. To verify the effectiveness of the proposed algorithm, we compared the IFCM-MS with five other advanced algorithms on the BRATS 2012 real brain tumor image dataset. Moreover, the results based on subjective evaluation and objective discussion indicated that the IFCM-MS outperformed the other fuzziness-based algorithms and provided an appreciable effect on the brain medical image segmentation. 

The IFCM-MS was designed mainly based on spatial information, whereas the integration of a specific mathematical theory can also overcome the shortcomings of the FCM. Therefore, we will continue the following works from this aspect. Moreover, the IFCM-MS was only centered on brain tumor MRI segmentation without processing the normal brain image. Further research can also be carried out on this type of medical image.

## Figures and Tables

**Table 1:** Dates of the ablation experiments.

Improvement Results	Accuracy	Precision	Recall	Specificity
Figure 2b	0.8659	0.8695	0.8624	0.9880
Figure 2c	0.8872	0.8627	0.9019	0.9914
Figure 2d	0.9616	0.9729	0.9412	0.9949
Figure 2e	**0.9623**	**0.9829**	**0.9519**	**0.9950**

**Table 2:** Average values of the ablation experiments (mean ± standard deviation). FCM Fuzzy C-means algorithm and IFS: intuitionistic fuzzy set.

Improvement Results	Accuracy	Precision	Recall	Specificity
Original FCM	0.8115 ± 0.0486	0.8112 ± 0.0583	0.8170 ± 0.0748	0.9874 ± 0.0063
FCM + IFS	0.8330 ± 0.0378	0.8108 ± 0.0542	0.8613 ± 0.0672	0.9903 ± 0.0062
FCM + IFS + Membership information transferring	0.9260 ± 0.0292	0.9341 ± 0.0558	0.9125 ± 0.0692	0.9929 ± 0.0055
FCM + IFS + Membership information transferring + Similarity measure	**0.9274** ± 0.0286	**0.9595** ± 0.0213	**0.9202** ± 0.0601	**0.9937** ± 0.0056

**Table 3:** Average values of the comparisons and the IFCM-MS (mean ± standard deviation). sFCM: spatial fuzzy C-means clustering algorithm, csFCM: conditional spatial fuzzy C-means clustering algorithm and FRFCM: FCM algorithm based on membership function filtering.

Algorithm	Intensity	Accuracy	Precision	Recall	Specificity
FCM [23]	0	0.9031 ± 0.0308	0.9707 ± 0.0302	0.8484 ± 0.0674	0.9894 ± 0.0061
	0.02	0.8115 ± 0.0486	0.8112 ± 0.0583	0.8170 ± 0.0748	0.9874 ± 0.0063
IFCM [24]	0	0.9097 ± 0.0295	0.9710 ± 0.0283	0.9005 ± 0.0599	0.9927 ± 0.0060
	0.02	0.8332 ± 0.0378	0.8108 ± 0.0542	0.8613 ± 0.0672	0.9903 ± 0.0062
sFCM [28]	0	0.9085 ± 0.0326	0.9694 ± 0.0350	0.8596 ± 0.0724	0.9893 ± 0.0069
	0.02	0.8352 ± 0.0493	0.8515 ± 0.0783	0.8279 ± 0.0785	0.9870 ± 0.0072
csFCM [25]	0	0.9043 ± 0.0325	0.9721 ± 0.0305	0.8499 ± 0.0714	0.9896 ± 0.006
	0.02	0.8924 ± 0.0362	0.9378 ± 0.0448	0.8545 ± 0.0610	0.9887 ± 0.0056
FRFCM [30]	0	0.9114 ± 0.0334	***0.9733*** ± 0.0304	0.8617 ± 0.0729	0.9908 ± 0.0052
	0.02	0.9105 ± 0.0328	**0.9734** ± 0.0307	0.8601 ± 0.0729	0.9904 ± 0.0065
IFCM-MS	0	***0.9279*** ± 0.0283	0.9458 ± 0.0219	***0.9172*** ± 0.0583	***0.9934*** ± 0.0055
	0.02	**0.9274** ± 0.0286	0.9595 ± 0.0213	**0.9202** ± 0.0601	**0.9937** ± 0.0056

## Data Availability

The dataset that support the findings of this study are available in (http://www.slicer.org/pages/Special:SlicerDownloads).
